# Supplementary material for: Medical relevance of protein-truncating variants across 337,205 individuals in the UK Biobank study
Source: Nat Commun. 2018 Apr 24;9:1612. doi: 10.1038/s41467-018-03910-9 (PMC5915386; doi:10.1038/s41467-018-03910-9)
Supplement: Supplementary file 1 — Supplementary Information [file 41467_2018_3910_MOESM1_ESM.docx]

**Supplementary Information**

**Medical relevance of protein-truncating variants across 337,205 individuals in the UK Biobank study**

**DeBoever et al.**

**Supplementary Figures**

**Supplementary Figure 1. gnomAD allele frequency comparison.**(a) PTV allele frequency and (b) log_10_ allele frequency among gnomAD non-Finnish Europeans (NFE) exome dataset and 337,205 UK Biobank (UKB) participants used in this study for all PTVs that could be matched between the two datasets. Small scatter points indicate PTVs that were filtered out for any reason. (c) PTV allele frequency and (d) log_10_ allele frequency among gnomAD non-Finnish Europeans exome dataset and 337,205 UK Biobank participants used in this study for PTVs that passed filtering and could be matched between the two datasets. (e) MAF histogram for all 18,228 polymorphic PTVs that passed filtering. (f) log_10_ MAF histogram for all 18,228 polymorphic PTVs that passed filtering. (g) MAF histogram for 17,765 PTVs with MAF<0.01. (h) MAF histogram for 17,065 PTVs with MAF<0.001.

**Supplementary Figure 2. Case numbers for phenotypes with at least 2,000 cases.** Number of cases for (a) cancers with more than 2,000 cases, (b) family history with more than 2,000 cases, (c) high confidence phenotypes with more than 20,000 cases, and (d) high confidence phenotypes with more than 2,000 cases but less than 20,000 cases. Bars are colored according to the number of cases identified from Hospital Episode Statistics (blue), questionnaire data (orange), or both (green).

**
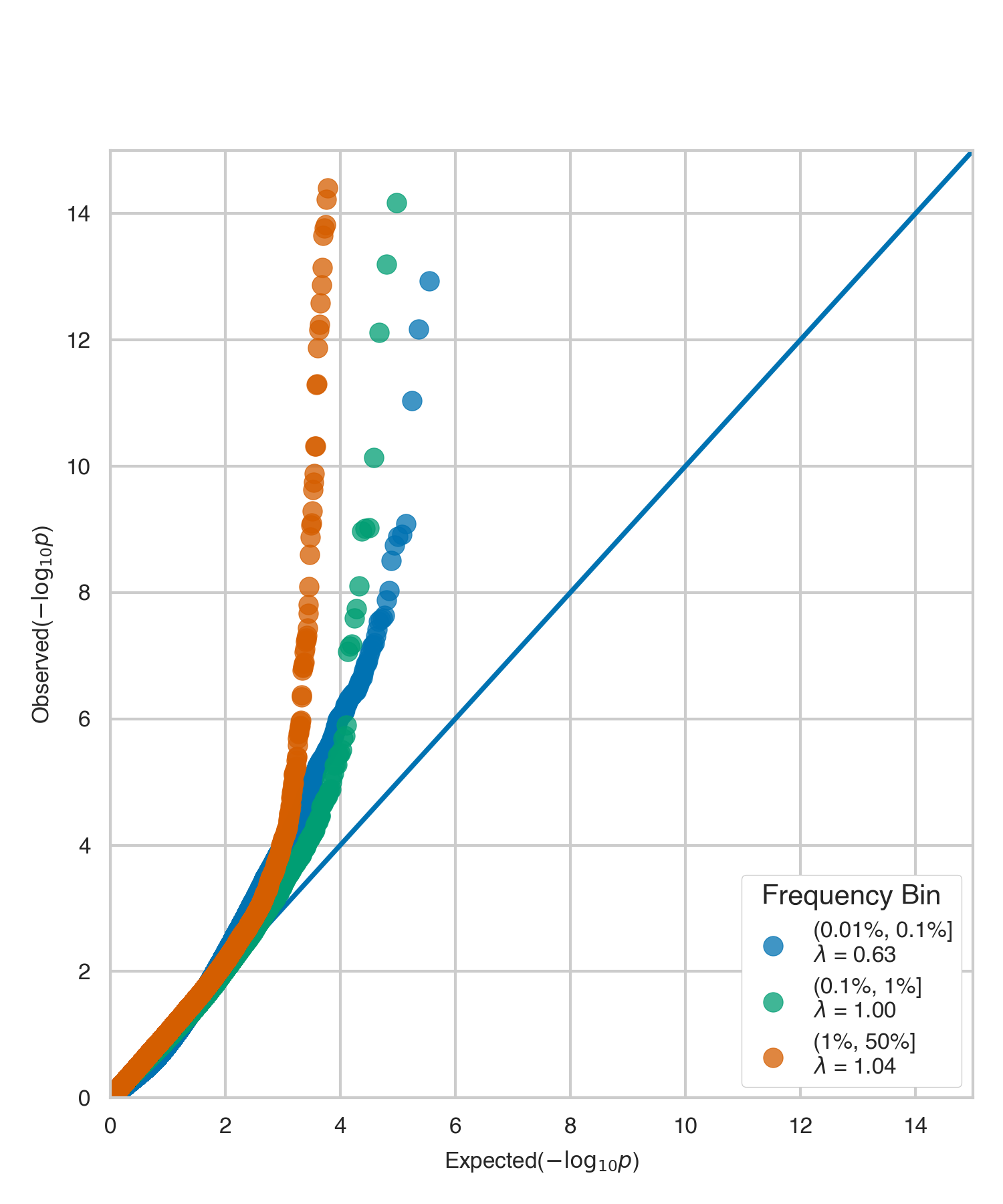
**

**Supplementay Figure 3. GWAS QQ plots.** QQ plots for single variant association analyses for 3,724 PTVs stratified into three minor allele frequency bins: (0.01%, 0.1%], (0.1%, 1%], (1%, 50%]. 26 associations with -log_10_ p-values greater than 14 are not shown.

**Supplementary Figure 4. *In vitro* experimental validation of NOL3 knockdown.** (a) *NOL3* mRNA expression measured 24h after treatment with *NOL3* siRNA or scramble negative control siRNA. (b) Activation of caspase 8, (c) DNA fragmentation, and expression of MAFbx (d) mRNA and (e) protein (quantification plot and original image) in NOL3 knockdown cells (NOL3si) and controls (Scramble) with or without electrical pulse stimulation (Stimulated or Unstimulated).

**Supplementary Figure 5. Linkage disequilibrium for PTVs and nearby variants.**Linkage disequilibrium between PTVs with significant associations and genotyped variants within 10kb (minimum LD 0.9). This figure includes PTVs with MAF > 1% for which conditional analysis identified a nearby variant that reduced the association p-value by at least one order of magnitude. For the PTV rs2884737 in VKORC1, we plotted variants with LD > 0.5. For rs2004640 in IRF5, we plotted variants with LD > 0.6. The variant ID of the PTV is bolded.

**
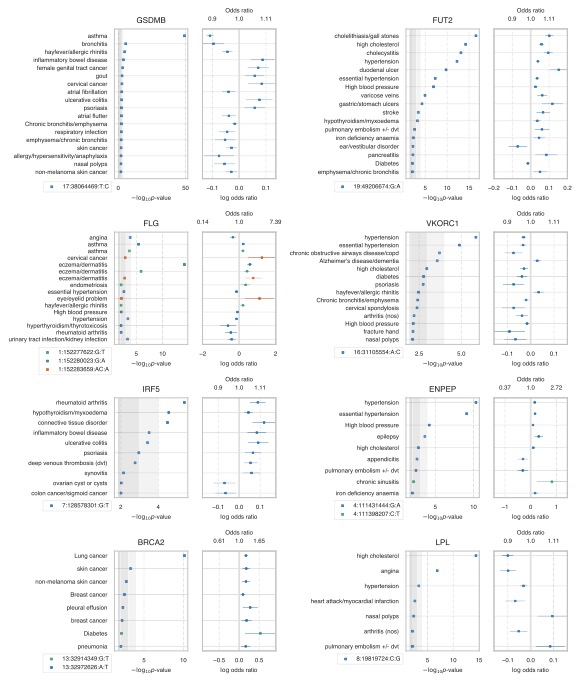
**

**
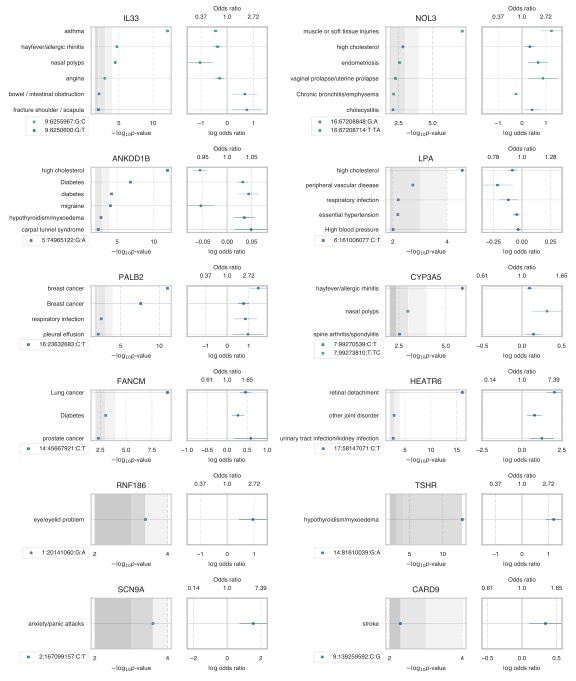
**

**Supplementary Figure 6. PheWAS results.**-log_10_ p-values and odds ratios for associations (p<0.01) for PTVs in 21 genes and 135 medical phenotypes. The gene for each plot is indicated above the p-value panel. *IFIH1*is plotted in Fig. 3.

**
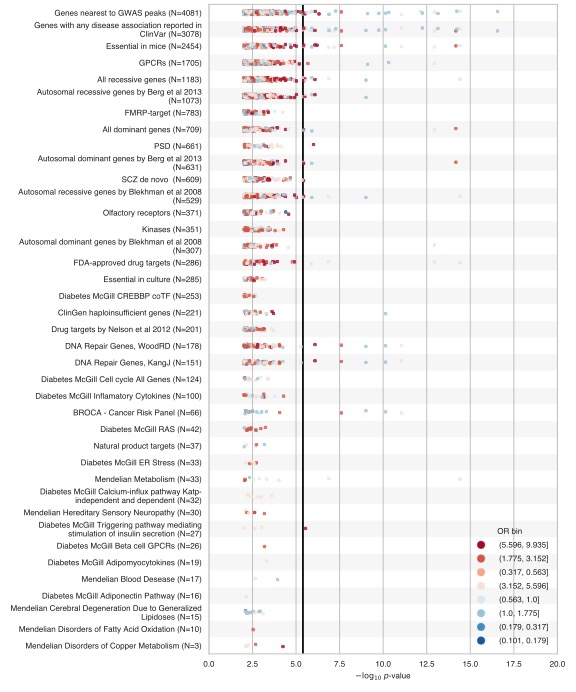
**

**Supplementary Figure 7. Gene set pheWAS.**-log10 p-value for associations with p<0.01 from our GWAS for PTVs in the genes in each gene set and 135 phenotypes with more than 2,000 cases. The black line indicates the nominal p-value cut-off for significance for the 74 associations identified in the initial GWAS. One association with p-value less than 10^-20^ is not plotted.

**Supplementary Figure 8. Case numbers for phenotypes with between 1,000 and 2,000 cases.**Number of cases for (a) cancers with more than 1,000 cases but less than than 2,000 cases and (b) high confidence phenotypes with more than 1,000 cases but less than 2,000 cases. Bars are colored according to the number of cases identified from Hospital Episode Statistics (blue), questionnaire data (orange), or both (green).

**
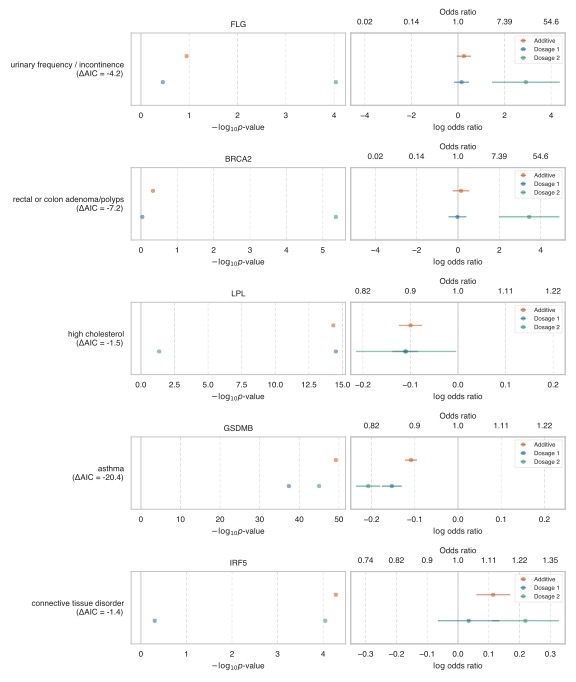
**

**Supplementary Figure 9. Non-additive associations.** The left panel shows the -log_10_ p-values for (blue) additive genetic model and (green, orange) non-additive genetic model. The right panel shows the estimated odds ratio and 95% confidence interval for (blue) additive genetic model and (green, orange) non-additive genetic model. The gene-phenotype association is labeled on the left with the difference in AIC between the additive and non-additive models. A more negative difference in AIC favors the non-additive model. *FUT2*is plotted in Fig. 4.

**Supplementary Tables**

**Supplementary Table 1. Genes with known protective associations.**We identified eight genes that did not have significant associations in our single-variant analysis (BY-adjusted p<0.05) but have been reported in the literature to protect against different diseases. We included these genes in our pheWAS and additivity analyses.

| Gene | Protects against | Reference |
| --- | --- | --- |
| *CARD9* | Crohn’s disease and/or ulcerative colitis | Rivas 2011, Rivas 2016 |
| *RNF186* | Crohn’s disease and/or ulcerative colitis | Rivas 2011, Rivas 2016 |
| *IL23R* | Crohn’s disease and/or ulcerative colitis | Rivas 2011, Rivas 2016 |
| *ANGPTL4* | coronary heart disease | Stitziel 2016, Musunuru 2017, Stitziel 2017 |
| *PCSK9* | coronary heart disease | Cohen 2005, Cohen 2006 |
| *LPA* | coronary heart disease | Lim 2014 |
| *APOC3* | coronary heart disease | Saleheen 2017 |
| *SCN9A* | pain | Nahorski 2015 |
