# Supplementary material for: Medical relevance of protein-truncating variants across 337,205 individuals in the UK Biobank study
Source: Nat Commun. 2018 Apr 24;9:1612. doi: 10.1038/s41467-018-03910-9 (PMC5915386; doi:10.1038/s41467-018-03910-9)
Supplement: Supplementary file 3 — Description of Additional Supplementary Files [file 41467_2018_3910_MOESM3_ESM.pdf]

Description of Additional Supplementary Files

File Name: Supplementary Data 1
Description: Number of genes for which homozygous loss-of-function variants were observed in ExAC (N=60,706) 9 , Icelanders (N=104,220) 7 , Pakistan Risk of Myocardial Infarction Study (PROMIS) (N=10,503) 13, UK Biobank (this study), and Pakistanis living in the UK 6 . The genes for ExAC and Icelanders were obtained from 6. The UK Biobank (MAF

File Name: Supplementary Data 2
Description: 206 medical phenotypes used in this study. Category indicates whether the phenotype was derived from family history questionnaire information (FH), diagnosis of a cancer (CA), or other disease (HC). See Methods and Supplementary Fig. 2,9 for more information.

File Name: Supplementary Data 3
Description: Significant associations from PTV and missense GWASs and pheWAS analysis. Variant IDs are from the UK Biobank data release. “gwas_protective” and “gwas_risk” tabs contain significant PTV associations (BY-adjusted p

File Name: Supplementary Data 4
Description: p-values for genetic effects for PTVs in MHC genes from our initial GWAS (“P_variant_gwas”) and from an analysis conditional on the HLA allele in the “HLA_subtype” column (“P_variant_conditional”). “P_subtype_conditional” contains the p-value for the association between the given HLA subtype and phenotype in the conditional analysis.

File Name: Supplementary Data 5
Description: Results from fitting additive and non-additive models of association for PTV carrier status (no PTVs, heterozygous knockout, or homozygous knockout) in 25 genes against 206 medical phenotypes with at least 1,000 cases. Columns that begin with “dosage1” and “dosage2” correspond to results for the non-additive model while columns that begin with “additive” correspond to the additive model. “aic_diff” is the AIC of the additive model subtracted from the AIC of the non-additive model.
